# Bis[3-(anthracen-9-yl)pentane-2,4-dionato-κ^2^ *O*,*O*′](*N*,*N*-di­methyl­formamide-κ*O*)[tris­(pyrazol-1-yl-κ*N* ^2^)hydroborato]europium(III)

**DOI:** 10.1107/S2056989022000676

**Published:** 2022-01-28

**Authors:** Elena A. Mikhalyova, Matthias Zeller, Jerry P. Jasinski, Manpreet Kaur, Anthony W. Addison

**Affiliations:** a L. V. Pisarzhevskii Institute of Physical Chemistry of the National Academy of Sciences of Ukraine, Prospekt Nauki 31, Kyiv, 03028, Ukraine; b Department of Chemistry, Purdue University, 560 Oval Drive, West Lafayette, IN, 47907, USA; c Department of Chemistry, Keene State College, Keene, NH 03435, USA; d Department of Chemistry, Drexel University, Philadelphia, PA 19104-2816, USA

**Keywords:** crystal structure, lanthanide complexes, luminescence, antenna ligands

## Abstract

The title compound, [Eu(C_9_H_10_BN_6_)(C_19_H_15_O_2_)_2_(C_3_H_7_NO)] or [TpEu(Anthracac)_2_(DMF)], was synthesized by reaction of a tris­(pyrazol­yl)borate (Tp^−^) Eu^3+^ complex with 3-(anthracen-9-yl)pentane-2,4-dione (HAnthracac) in the presence of tri­ethyl­amine. In the title compound, Eu^3+^ is located in an octa­vertex square-pyramidal coordination environment. In the two Anthracac^−^ ligands, the anthracene and nearly planar acetyl­acetonate fragments are almost orthogonal. Neighboring mol­ecules of TpEu(Anthracac)_2_(DMF) are connected in the crystal by inter­molecular van der Waals inter­actions, while π-stacking inter­actions are limited to the edges of two anthracene rings.

## Abstract 2

The title Eu complex, featuring both Anthracac and tris­pyrazolyl­hydro­borato ligands, exhibits an octa­vertex square-pyramidal coordination environment.

## Chemical context

Lanthanide complexes find numerous applications as, for example, luminescent materials, markers, security inks, components of lasers, light-emitting diodes, and many others (Bünzli, 2017 ▸; Venturini Filho *et al.*, 2018 ▸; Khullar *et al.*, 2019 ▸; Bünzli, 2019 ▸). This variety of uses relies in large parts on the electronic structure of the *Ln* ^3+^ ions, which leads to electronic transitions occurring between *f*-orbitals, providing them with unique luminescence characteristics, including high color purity and exact reproducibility of the emitted light color (Sarkar *et al.*, 2019 ▸; Wang, Pu *et al.*, 2019 ▸; Wang, Zhao *et al.*, 2019 ▸). In spite of these advantages, the electronic structure of *Ln* ^3+^ ions causes the luminescence to be of low intensity due to the forbidden nature of *f–f* electronic transitions (Bünzli, 2017 ▸; Zhang *et al.*, 2020 ▸; Wang, Zhao *et al.*, 2019 ▸), hence the weak absorbance of the exciting radiation. This feature is usually evaded by using organic ‘antenna’ ligands, which are capable of absorbing exciting radiation and transferring the gained energy to the *Ln* ^3+^ ions (Bünzli, 2017 ▸; Carneiro Neto *et al.*, 2019 ▸; Aulsebrook *et al.*, 2018 ▸). Recently, it was shown (Mikhalyova *et al.*, 2017 ▸; Gheno *et al.*, 2014 ▸; Mikhalyova *et al.*, 2020 ▸; Bortoluzzi *et al.*, 2012 ▸) that tris­(pyrazol­yl)borate anions are efficient antenna ligands for Tb^3+^ and Eu^3+^, both exhibiting emission in the visible range. Anions of *β*-diketones with different substituents are also well-known antenna ligands (Wang, Zhao *et al.*, 2019 ▸; Nehra *et al.*, 2022 ▸). To increase the extinction coefficients of the ligands, it can be of advantage to add a large conjugated moiety to their structure. Recently it was found by us (Kandel *et al.*, 2017 ▸; Mikhalyova *et al.*, 2017 ▸), that the combination of several antenna ligands in one compound can have complex and unpredictable effects on its luminescence characteristics, which also depend on the mol­ecular and crystal structure details of the complex. Thus, for this work, an Eu^3+^ complex with two types of antenna ligands, *i.e.* tris­(pyrazol­yl)borate (Tp^−^) and 3-(anthracen-9-yl)pentane-2,4-dionate (Anthracac^−^), of the composition TpEu(Anthracac)_2_(DMF) was obtained and its mol­ecular and crystal structures were studied. 
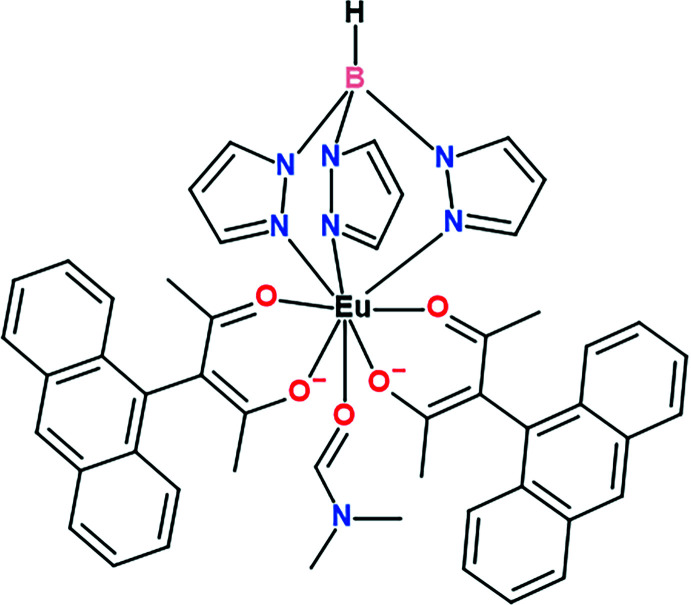


## Structural commentary

**Figure 1:**
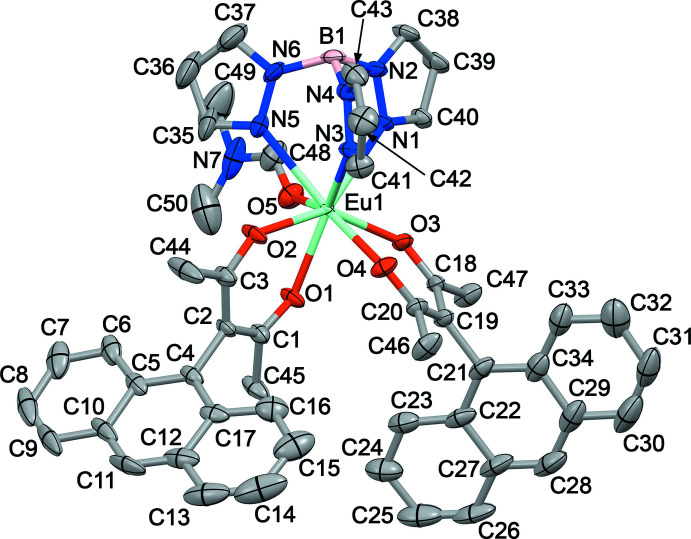
The mol­ecular structure of the title compound. Atomic displacement ellipsoids are drawn at the 50% probability level. H atoms are omitted for clarity of presentation.

**Figure 2:**
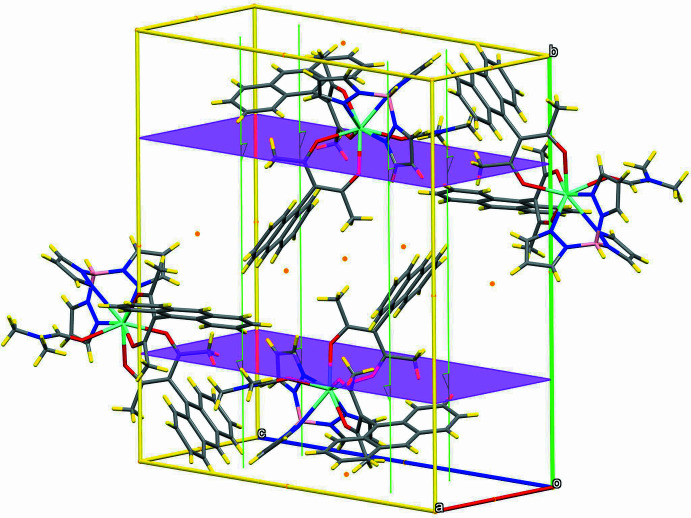
Stick diagram of a unit-cell view with symmetry elements: inversion centers (orange), glide planes (violet) and screw axes (green).

**Figure 3:**
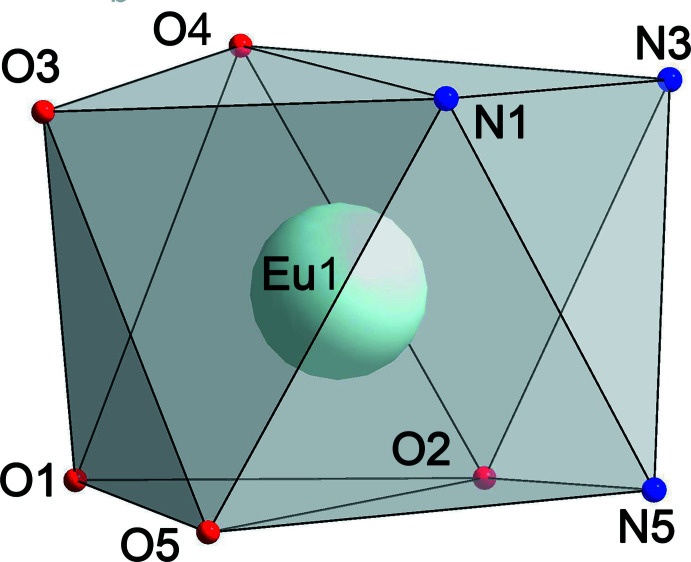
The geometry of the Eu^3+^ coordination polyhedron.

The title compound is a neutral metal-containing complex and crystallizes in the monoclinic *P*2_1_/*n* space group with only one mol­ecule in the asymmetric unit (Fig. 1 ▸). The unit cell contains two mol­ecules of each enanti­omer, whose crystallographic positions are related by the inversion centers, glide planes and screw axes (Fig. 2 ▸). The asymmetric unit consists of the Eu^3+^ ion surrounded by one Tp^−^ and two Anthracac^−^ ligands and one di­methyl­formamide mol­ecule. Of these ligands, the Tp^−^ is coordinated tridentately, donating three N atoms to the coordination polyhedron, while each Anthracac^−^ acts as a bidentate O ligand, donating a combined four O atoms. The DMF mol­ecule acts as a unidentate O donor. As is typical for lanthanide ions with seven, eight or nine coordinating atoms, the assignment of the coordination geometry carries some ambiguity. Several different criteria have been proposed to define the shape of such a coordination polyhedron. Use of the *Shape 2.1* software (Casanova *et al.*, 2005 ▸; Alvarez *et al.*, 2005 ▸), indicates that the Eu^3+^ ion in the title compound is an octa­vertex with a slightly distorted square-anti­prismatic geometry (Fig. 3 ▸), with a mean angle between the capping and basal square planes of the coordination polyhedron of 0.75 (8)°. According to the Lippard & Russ (1968 ▸) criterion, the angle between the body-diagonal trapezoids for the title compound, ω, is 88.24 (7)°, which is closer to the angle for a dodeca­hedron (90.0°) than a square anti­prism (79.3°). A more accurate criterion is the one proposed by Porai-Koshits and Aslanov (1972 ▸) based on the angles, δ, between pairs of faces inter­secting along the edges connecting the vertices where the five edges inter­sect. The respective angles for the complex here are 6.6 (1), 8.9 (1), 43.3 (1), and 49.7 (1)° and the degrees of non-planarity of the diagonal trapezoids, φ, are 18.81 (9) and 19.74 (1)°. From these criteria, the δ angles are closer to those of an idealized square anti­prism, yet the φ angles correspond to those of a bicapped trigonal prism. Thus, three different criteria define three different polyhedra and among these criteria, only the δ-based one agrees with the assignment using the *Shape 2.1* software.

The lengths for Eu-donor atom bonds are listed in Table 1 ▸ and these are in the usual range for compounds with similar ligands (Mikhalyova *et al.*, 2020 ▸; Lawrence *et al.*, 2001 ▸; Dei *et al.*, 2000 ▸).

Regarding the geometrical features of the ligands, it should be noted that the planar anthracene moiety and the nearly planar acetyl­acetonate fragment are almost orthogonal to each other in each Anthracac^−^ ligand, subtending dihedral angles of 87.84 (7) and 79.98 (7)°. This is due to the presence of the CH_3_ groups, which prevent rotation of the anthracenyl fragments along the C2—C4 and C19—C21 bonds.

## Supra­molecular features

**Figure 4:**
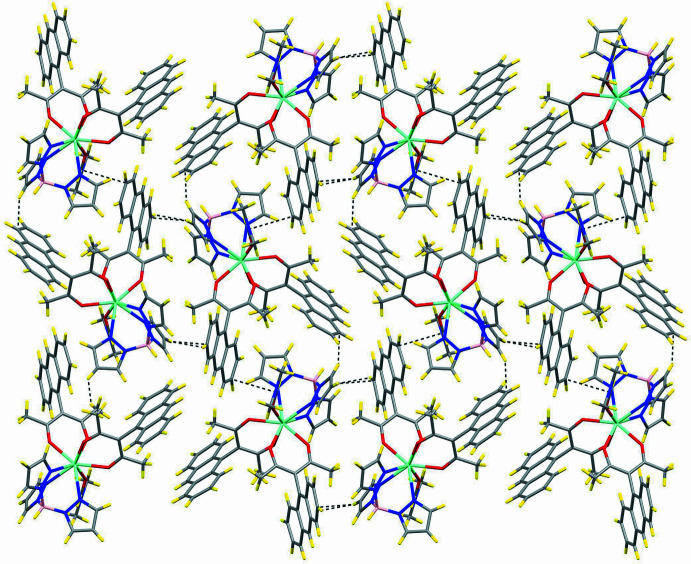
Packing view along the *a*-axis (see also Fig. S3).

The crystal packing of the title compound consists of separate neutral mol­ecules. Several short contacts are observed (Table 2 ▸), but none of these exhibit the typical characteristics of directional attractive inter­actions, *i.e.* they are not hydrogen bonds or C—H⋯π inter­actions. It thus can be said that these mol­ecules are organized in the lattice predominantly by inter­molecular van der Waals or dispersion inter­actions (Fig. 4 ▸, Table 2 ▸).

**Figure 5:**
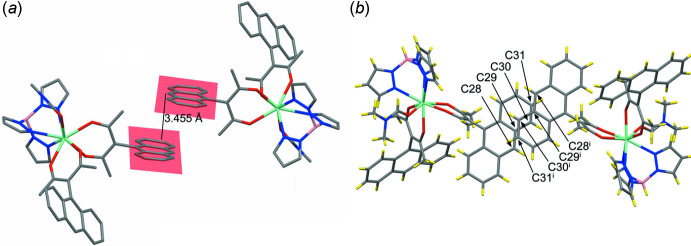
(*a*) View of the π–π stacking inter­action observed for one of the phenyl­ene groups of the anthracene fragments and (*b*) a view of the same, perpendicular to the planes of the anthracenyl (C21—C34) fragments [symmetry code: (i) 2 − *x*, 1 − *y*, 1 − *z*]. Other occurrences of parallel (but not stacked) anthracenyl units are shown in Figs. S1, S2 and S4.

π-Stacking inter­actions play no dominant role in this structure. For one of the anthracene fragments (C4–C16) no π–π stacking inter­actions are observed at all. For the other anthracenyl group (C21–C34) one π-inter­action is present, but it is limited to part of one of the outer phenyl­ene groups, C29–C34, which is π-stacked with its inversion-related counterpart [symmetry code: (i) 2 − *x*, 1 − *y*, 1 − *z*], with a centroid-to-centroid distance of 3.958 (8) Å (Fig. 5 ▸). The remainder of the anthracenyl group does not participate in the π–π stacking inter­action; for the entire anthracene moiety (C21–C34) the distance between the centroids is 6.006 (8) Å. The distance between inversion-related mean planes (C21–C34 and C21^i^–C34^i^) is 3.455 Å, indicating a medium strength stacking inter­action (Fig. 5 ▸).

## Database survey

The Cambridge Structural Database (CSD, version 5.41, updates till Aug 2020; Groom *et al.*, 2016 ▸) contains just one crystal structure with an *Ln* ^3+^ ion surrounded by two *β*-diketonate anions and one tris­(pyrazol­yl)borate ligand, namely, bis­(1,3-diphenyl-1,3-propane­dionato-*O*,*O*′){hydro­tris­[3-(2-pyrid­yl)pyrazol-1-yl]borato}praseodymium(III) (FOLZUC; Davies *et al.*, 2005 ▸). However, in this compound the Pr^3+^ ion is deca­coord­inate owing to the presence of 2-pyridyl substituents in the tris­(pyrazol­yl)borate ligand, so a direct comparison of the coordination geometries of this and the title compound is not possible.

Fragments containing one *Ln* ion surrounded by at least one *β*-diketonate anion and one tris­(pyrazol­yl)borate ligand encompass 34 entities (including FOLZUC). Most of them (28), contain eight-coordinate lanthanide ions with two tris(pyrazol­yl)borate ligands and one *β*-diketonate anion: DULWEP, DULWIT, DULWOZ, DULWUF, DULXAM, DULXEQ, DULXIU, DULXOA, DULXUG, DULYAN, DULYER, DULYIV, DULYOB, DULYUH, DULZAO, DULZES, DULZIW, DULZOC, DULZUI and DUMDAT (all Mikhalyova *et al.*, 2020 ▸); ESUHOP (Galler *et al.*, 2004 ▸); GIFCUT, GIFDAA (Moss *et al.*, 1988 ▸); GIFCUT10, GIFDAA10 (Moss *et al.*, 1989 ▸); KIFKUI (Guégan *et al.*, 2018 ▸); XICHIA (Lawrence *et al.*, 2001 ▸). Again, the coordination environment of these compounds and the title one cannot be directly compared. One of the compounds, FOLZUC, is discussed above and another, [tris­(3-*t*-butyl-5-methyl­pyrazol­yl)hydro­borato](2,2,6,6-tetra­methyl­heptane-3,5-dionato)ytter­bium(II) (ESUJIL; Morissette *et al.*, 2004 ▸) is a neutral mol­ecule of Yb^2+^. Four entities are complexes with salicyl­aldehyde derivatives [JAJRAO (Onishi *et al.*, 2004 ▸), QIDGAL, QIDGAL01 (Onishi *et al.*, 1999 ▸), and ZUCCIJ (Lawrence *et al.*, 1996 ▸)], which are also *β*-diketonate anions, but, again, compounds with these anions contain eight-coord­inate *Ln* ^3+^ ions.

Only three metal-containing structures were found with 3-naphthyl or 3-anthracenyl substituents. The inter­planar angles for acetyl­acetonate *vs* aryl fragments are 86.4° for [3-(1′-naphth­yl)pentane-2,4-dionato][tris­(2-amino­eth­yl)amine]­cobalt(III) bis­(tetra­fluoro­borate) dihydrate, 87.1° for [3-(2′,4′-di­nitro-1′-naphth­yl)pentane-2,4-dionato][tris­(2-amino­eth­yl)amine]­cobalt(III) dibromide (BEYTEE and BIMLUE, respectively; Nakano & Sato, 1982 ▸) and 83.5° for [3-(9′-anth­r­yl)acetyl­acetonato]chlorido­(1,4,7-trimethyl-1,4,7-tri­aza­cyclo­nona­ne)iron(III) perchlorate mesitylene solvate (NUCZUG; Müller *et al.*, 1998 ▸). These angles are in the same range as for the title compound.

## Synthesis and crystallization

The starting Tp_2_EuCl complex was obtained by reaction of TpTl with EuCl_3_·6H_2_O in methanol (Kandel *et al.*, 2017 ▸). Then, 307 mg (0.50 mmol) of Tp_2_EuCl and 138 mg (0.50 mmol) of HAnthracac were dissolved in 15 mL of methyl­ene chloride, followed by the addition of 0.15 mL of tri­ethyl­amine. After the solution had been stirred for 1 h, the reaction mixture was filtered and the filtrate was evaporated under reduced pressure (rotavapor). The resulting residue was washed with water and dried in a vacuum desiccator over P_2_O_5_. The crude product was recrystallized by slow diffusion of methyl *t*-butyl ether into a DMF solution of the compound. The title compound was obtained as orange prismatic crystals (25 mg, yield 10%).

## Refinement

Crystal data, data collection and structure refinement details are summarized in Table 3 ▸. C—H bond distances were constrained to 0.95 Å for aromatic and alkene C—H moieties, and to 0.98 Å for CH_3_ moieties. The B—H bond distance was constrained to 1.00 Å. *U*_iso_(H) values were set to *kU*_eq_(C) where *k* = 1.5 for CH_3_ and 1.2 for C—H units.

## Supplementary Material

Crystal structure: contains datablock(s) I. DOI: 10.1107/S2056989022000676/yy2007sup1.cif

Structure factors: contains datablock(s) I. DOI: 10.1107/S2056989022000676/yy2007Isup2.hkl

Supporting information file. DOI: 10.1107/S2056989022000676/yy2007sup3.pdf

CCDC reference: 2143190

Additional supporting information:  crystallographic information; 3D view; checkCIF report

## Figures and Tables

**Table 1:** Selected bond lengths (Å)

Eu1—O1	2.351 (3)	Eu1—O5	2.417 (3)
Eu1—O2	2.313 (3)	Eu1—N1	2.582 (3)
Eu1—O3	2.344 (3)	Eu1—N3	2.555 (3)
Eu1—O4	2.340 (3)	Eu1—N5	2.573 (3)

**Table 2:** Selected inter­molecular inter­atomic distances (Å)

C8⋯C15^i^	3.258 (8)	C8⋯H36^ii^	2.698
H8⋯C15^i^	2.817	H37⋯C26^iii^	2.830
H50*C*⋯N3^i^	2.680	C48⋯C14^iii^	3.159 (8)
H50*C*⋯C41^i^	2.718		

Symmetry codes: (i) −1 + *x*, *y*, *z*; (ii) *x*, 2 − *y*, 1 − *z*; (iii) −{1\over 2} + *x*, {3\over 2} − *y*, −{1\over 2} + *z*.

**Table 3:** Experimental details

Crystal data	
Chemical formula	[Eu(C_9_H_10_BN_6_)(C_19_H_15_O_2_)_2_(C_3_H_7_NO)]
*M*_r_	988.71
Crystal system, space group	Monoclinic, *P*2_1_/*n*
Temperature (K)	173
*a*, *b*, *c* (Å)	9.3728 (3), 22.5555 (7), 22.0840 (6)
β (°)	96.314 (3)
*V* (Å^3^)	4640.4 (2)
*Z*	4
Radiation type	Cu *K*α
μ (mm^−1^)	10.11
Crystal size (mm)	0.48 × 0.18 × 0.12

Data collection	
Diffractometer	Rigaku Oxford Diffraction Gemini Eos
Absorption correction	Multi-scan (*CrysAlis PRO*; Rigaku OD, 2015 ▸)
*T*_min_, *T*_max_	0.163, 1.000
No. of measured, independent and observed [*I* > 2σ(*I*)] reflections	19671, 8850, 7261
*R*_int_	0.048
(sin θ/λ)_max_ (Å^−1^)	0.615

Refinement	
*R*[*F* ^2^ > 2σ(*F* ^2^)], *wR*(*F* ^2^), *S*	0.042, 0.106, 1.03
No. of reflections	8850
No. of parameters	583
H-atom treatment	H-atom parameters constrained
Δρ_max_, Δρ_min_ (e Å^−3^)	1.09, −1.25

Computer programs: *CrysAlis PRO* (Rigaku OD, 2015 ▸), *SHELXT* (Sheldrick, 2015*a* ▸), *SHELXL* (Sheldrick, 2015*b* ▸), *OLEX2* (Dolomanov *et al.*, 2009 ▸), *Mercury* (Macrae *et al.*, 2020 ▸), and *publCIF* (Westrip, 2010 ▸).

## supplementary crystallographic information

### Crystal data

**Table:**

[Eu(C_9_H_10_BN_6_)(C_19_H_15_O_2_)_2_(C_3_H_7_NO)]	*F*(000) = 2016
*M**_r_* = 988.71	*D*_x_ = 1.415 Mg m^−^^3^
Monoclinic, *P*2_1_/*n*	Cu *K*α radiation, λ = 1.54184 Å
*a* = 9.3728 (3) Å	Cell parameters from 8094 reflections
*b* = 22.5555 (7) Å	θ = 4.0–71.4°
*c* = 22.0840 (6) Å	µ = 10.11 mm^−^^1^
β = 96.314 (3)°	*T* = 173 K
*V* = 4640.4 (2) Å^3^	Prism, orange
*Z* = 4	0.48 × 0.18 × 0.12 mm

### Data collection

**Table:**

Rigaku Oxford Diffraction Gemini Eos diffractometer	8850 independent reflections
Radiation source: fine-focus sealed X-ray tube, Enhance (Cu) X-ray Source	7261 reflections with *I* > 2σ(*I*)
Graphite monochromator	*R*_int_ = 0.048
Detector resolution: 16.0416 pixels mm^-1^	θ_max_ = 71.4°, θ_min_ = 3.9°
ω scans	*h* = −11→11
Absorption correction: multi-scan (CrysAlisPro; Rigaku OD, 2015)	*k* = −27→17
*T*_min_ = 0.163, *T*_max_ = 1.000	*l* = −26→24
19671 measured reflections	

### Refinement

**Table:**

Refinement on *F*^2^	Primary atom site location: dual
Least-squares matrix: full	Hydrogen site location: inferred from neighbouring sites
*R*[*F*^2^ > 2σ(*F*^2^)] = 0.042	H-atom parameters constrained
*wR*(*F*^2^) = 0.106	*w* = 1/[σ^2^(*F*_o_^2^) + (0.0592*P*)^2^] where *P* = (*F*_o_^2^ + 2*F*_c_^2^)/3
*S* = 1.03	(Δ/σ)_max_ = 0.004
8850 reflections	Δρ_max_ = 1.09 e Å^−^^3^
583 parameters	Δρ_min_ = −1.25 e Å^−^^3^
0 restraints	

### Special details

**Table:**

Geometry. All esds (except the esd in the dihedral angle between two l.s. planes) are estimated using the full covariance matrix. The cell esds are taken into account individually in the estimation of esds in distances, angles and torsion angles; correlations between esds in cell parameters are only used when they are defined by crystal symmetry. An approximate (isotropic) treatment of cell esds is used for estimating esds involving l.s. planes.

### Fractional atomic coordinates and isotropic or equivalent isotropic displacement parameters (Å^2^)

**Table:**

	*x*	*y*	*z*	*U*_iso_*/*U*_eq_	
Eu1	0.52702 (2)	0.81156 (2)	0.43393 (2)	0.01770 (8)	
O1	0.4093 (3)	0.77371 (12)	0.51430 (12)	0.0316 (6)	
O2	0.4941 (4)	0.88721 (12)	0.50175 (13)	0.0396 (8)	
O3	0.5692 (3)	0.70993 (11)	0.42273 (11)	0.0263 (6)	
O4	0.7264 (3)	0.78408 (11)	0.50130 (13)	0.0317 (6)	
O5	0.2953 (3)	0.77901 (14)	0.38675 (14)	0.0383 (7)	
N1	0.5855 (4)	0.79579 (14)	0.32351 (13)	0.0248 (7)	
N2	0.6057 (4)	0.83970 (15)	0.28333 (14)	0.0294 (7)	
N3	0.7242 (4)	0.88341 (14)	0.41029 (14)	0.0266 (7)	
N4	0.7259 (4)	0.91415 (14)	0.35743 (14)	0.0268 (7)	
N5	0.4131 (4)	0.89911 (15)	0.37142 (16)	0.0331 (8)	
N6	0.4623 (4)	0.92238 (15)	0.32156 (16)	0.0361 (8)	
N7	0.0779 (5)	0.7911 (2)	0.3344 (3)	0.0597 (14)	
C1	0.3798 (4)	0.79077 (17)	0.56538 (18)	0.0266 (8)	
C2	0.4045 (5)	0.84859 (17)	0.58988 (17)	0.0269 (8)	
C3	0.4583 (5)	0.89398 (18)	0.55457 (19)	0.0339 (10)	
C4	0.3751 (5)	0.86189 (16)	0.65368 (18)	0.0298 (9)	
C5	0.2383 (5)	0.88156 (16)	0.6657 (2)	0.0345 (10)	
C6	0.1212 (5)	0.88699 (18)	0.6203 (2)	0.0424 (11)	
H6	0.1357	0.8791	0.5792	0.051*	
C7	−0.0128 (6)	0.9033 (2)	0.6333 (3)	0.0591 (16)	
H7	−0.0900	0.9063	0.6018	0.071*	
C8	−0.0347 (9)	0.9156 (2)	0.6950 (4)	0.079 (3)	
H8	−0.1274	0.9266	0.7046	0.094*	
C9	0.0737 (9)	0.9117 (2)	0.7394 (3)	0.071 (2)	
H9	0.0562	0.9201	0.7800	0.085*	
C10	0.2147 (7)	0.89522 (18)	0.7277 (2)	0.0496 (15)	
C11	0.3259 (8)	0.8903 (2)	0.7737 (2)	0.0584 (18)	
H11	0.3095	0.9007	0.8140	0.070*	
C12	0.4595 (7)	0.87091 (19)	0.7630 (2)	0.0501 (14)	
C13	0.5756 (10)	0.8643 (2)	0.8103 (3)	0.072 (2)	
H13	0.5615	0.8757	0.8506	0.086*	
C14	0.7041 (10)	0.8424 (3)	0.7996 (3)	0.084 (3)	
H14	0.7790	0.8385	0.8320	0.101*	
C15	0.7271 (7)	0.8252 (3)	0.7400 (3)	0.0683 (19)	
H15	0.8169	0.8088	0.7328	0.082*	
C16	0.6219 (6)	0.8320 (2)	0.6926 (2)	0.0471 (12)	
H16	0.6406	0.8211	0.6527	0.056*	
C17	0.4842 (5)	0.85520 (18)	0.70201 (19)	0.0360 (10)	
C18	0.6316 (5)	0.66956 (16)	0.45555 (16)	0.0250 (8)	
C19	0.7273 (4)	0.67949 (16)	0.50837 (16)	0.0253 (8)	
C20	0.7690 (4)	0.73674 (18)	0.52778 (17)	0.0271 (8)	
C21	0.7840 (5)	0.62631 (17)	0.54511 (18)	0.0353 (10)	
C22	0.6957 (6)	0.59931 (18)	0.58541 (19)	0.0417 (12)	
C23	0.5594 (6)	0.6217 (2)	0.5950 (2)	0.0448 (12)	
H23	0.5257	0.6567	0.5741	0.054*	
C24	0.4744 (7)	0.5949 (3)	0.6332 (2)	0.0580 (15)	
H24	0.3843	0.6118	0.6394	0.070*	
C25	0.5201 (10)	0.5420 (3)	0.6635 (3)	0.075 (2)	
H25	0.4595	0.5229	0.6892	0.090*	
C26	0.6484 (9)	0.5188 (2)	0.6562 (2)	0.0654 (19)	
H26	0.6768	0.4828	0.6765	0.079*	
C27	0.7442 (7)	0.5466 (2)	0.6185 (2)	0.0503 (14)	
C28	0.8772 (7)	0.5250 (2)	0.6124 (2)	0.0587 (17)	
H28	0.9088	0.4905	0.6347	0.070*	
C29	0.9690 (7)	0.5512 (2)	0.5747 (2)	0.0540 (15)	
C30	1.1070 (8)	0.5289 (3)	0.5678 (3)	0.074 (2)	
H30	1.1411	0.4948	0.5903	0.089*	
C31	1.1914 (8)	0.5550 (3)	0.5299 (3)	0.071 (2)	
H31	1.2842	0.5391	0.5269	0.085*	
C32	1.1462 (7)	0.6054 (3)	0.4942 (3)	0.0692 (19)	
H32	1.2067	0.6229	0.4674	0.083*	
C33	1.0122 (6)	0.6281 (2)	0.4999 (2)	0.0511 (13)	
H33	0.9806	0.6620	0.4764	0.061*	
C34	0.9201 (6)	0.6030 (2)	0.5391 (2)	0.0434 (12)	
C35	0.2815 (6)	0.9201 (2)	0.3731 (3)	0.0520 (13)	
H35	0.2225	0.9118	0.4043	0.062*	
C36	0.2421 (8)	0.9556 (3)	0.3235 (4)	0.076 (2)	
H36	0.1529	0.9753	0.3135	0.091*	
C37	0.3605 (7)	0.9565 (3)	0.2915 (3)	0.0662 (17)	
H37	0.3686	0.9774	0.2547	0.079*	
C38	0.6116 (6)	0.8170 (2)	0.22766 (18)	0.0374 (10)	
H38	0.6252	0.8389	0.1920	0.045*	
C39	0.5948 (5)	0.7566 (2)	0.23078 (18)	0.0373 (10)	
H39	0.5933	0.7287	0.1985	0.045*	
C40	0.5805 (5)	0.74553 (18)	0.29162 (17)	0.0282 (8)	
H40	0.5688	0.7072	0.3083	0.034*	
C41	0.8332 (5)	0.9041 (2)	0.4480 (2)	0.0384 (10)	
H41	0.8576	0.8906	0.4886	0.046*	
C42	0.9064 (6)	0.9479 (2)	0.4203 (2)	0.0476 (12)	
H42	0.9882	0.9695	0.4372	0.057*	
C43	0.8344 (6)	0.95314 (19)	0.3629 (2)	0.0410 (11)	
H43	0.8576	0.9799	0.3323	0.049*	
C44	0.4746 (8)	0.9563 (2)	0.5808 (2)	0.067 (2)	
H44A	0.5506	0.9772	0.5623	0.101*	
H44B	0.4998	0.9540	0.6250	0.101*	
H44C	0.3839	0.9778	0.5720	0.101*	
C45	0.3115 (6)	0.74539 (18)	0.6032 (2)	0.0405 (11)	
H45A	0.2855	0.7102	0.5783	0.061*	
H45B	0.2251	0.7623	0.6177	0.061*	
H45C	0.3796	0.7342	0.6382	0.061*	
C46	0.8711 (6)	0.7442 (2)	0.5854 (2)	0.0445 (12)	
H46A	0.9668	0.7302	0.5782	0.067*	
H46B	0.8363	0.7211	0.6183	0.067*	
H46C	0.8762	0.7862	0.5968	0.067*	
C47	0.5960 (6)	0.60688 (18)	0.43470 (19)	0.0394 (11)	
H47A	0.5374	0.5879	0.4633	0.059*	
H47B	0.6850	0.5844	0.4333	0.059*	
H47C	0.5424	0.6078	0.3940	0.059*	
C48	0.2187 (6)	0.7920 (2)	0.3402 (3)	0.0481 (12)	
H48	0.2645	0.8035	0.3057	0.058*	
C49	−0.0009 (10)	0.8083 (3)	0.2768 (4)	0.106 (4)	
H49A	0.0668	0.8210	0.2485	0.158*	
H49B	−0.0658	0.8412	0.2835	0.158*	
H49C	−0.0568	0.7745	0.2594	0.158*	
C50	−0.0025 (8)	0.7747 (4)	0.3832 (4)	0.094 (3)	
H50A	0.0636	0.7644	0.4192	0.141*	
H50B	−0.0630	0.7404	0.3709	0.141*	
H50C	−0.0632	0.8079	0.3929	0.141*	
B1	0.6109 (6)	0.9057 (2)	0.3029 (2)	0.0331 (11)	
H1	0.6336	0.9311	0.2681	0.040*	

### Atomic displacement parameters (Å^2^)

**Table:**

	*U*^11^	*U*^22^	*U*^33^	*U*^12^	*U*^13^	*U*^23^
Eu1	0.02659 (12)	0.01614 (10)	0.01118 (10)	0.00364 (9)	0.00566 (7)	−0.00032 (7)
O1	0.0516 (18)	0.0232 (13)	0.0240 (13)	−0.0069 (12)	0.0212 (13)	−0.0064 (10)
O2	0.079 (2)	0.0166 (12)	0.0292 (14)	−0.0018 (14)	0.0342 (15)	−0.0044 (10)
O3	0.0391 (15)	0.0230 (12)	0.0160 (11)	0.0072 (11)	−0.0003 (11)	0.0018 (10)
O4	0.0422 (17)	0.0186 (12)	0.0318 (14)	−0.0022 (12)	−0.0072 (13)	0.0049 (11)
O5	0.0329 (16)	0.0390 (16)	0.0406 (17)	−0.0004 (13)	−0.0067 (14)	0.0018 (13)
N1	0.0393 (19)	0.0235 (15)	0.0127 (13)	0.0060 (13)	0.0075 (13)	−0.0013 (11)
N2	0.046 (2)	0.0281 (17)	0.0149 (14)	0.0025 (15)	0.0055 (14)	0.0022 (12)
N3	0.0397 (19)	0.0229 (14)	0.0178 (14)	−0.0003 (14)	0.0060 (14)	0.0029 (12)
N4	0.0397 (19)	0.0201 (14)	0.0225 (15)	−0.0010 (14)	0.0123 (14)	−0.0017 (12)
N5	0.037 (2)	0.0259 (16)	0.0366 (19)	0.0130 (15)	0.0052 (16)	0.0034 (14)
N6	0.050 (2)	0.0284 (17)	0.0277 (17)	0.0182 (16)	−0.0042 (16)	0.0058 (13)
N7	0.039 (2)	0.041 (2)	0.094 (4)	0.0157 (19)	−0.021 (3)	−0.021 (2)
C1	0.033 (2)	0.0217 (17)	0.0267 (18)	−0.0019 (15)	0.0119 (16)	−0.0023 (15)
C2	0.040 (2)	0.0227 (17)	0.0206 (17)	0.0004 (16)	0.0159 (16)	−0.0031 (14)
C3	0.056 (3)	0.0205 (18)	0.028 (2)	−0.0003 (18)	0.019 (2)	−0.0025 (15)
C4	0.048 (2)	0.0183 (17)	0.0268 (19)	−0.0071 (17)	0.0188 (18)	−0.0059 (14)
C5	0.054 (3)	0.0143 (16)	0.040 (2)	−0.0052 (17)	0.028 (2)	−0.0038 (15)
C6	0.047 (3)	0.0206 (18)	0.064 (3)	−0.0014 (19)	0.027 (2)	−0.0067 (19)
C7	0.050 (3)	0.024 (2)	0.108 (5)	0.000 (2)	0.028 (3)	−0.001 (3)
C8	0.092 (5)	0.022 (2)	0.140 (7)	0.009 (3)	0.090 (5)	0.010 (3)
C9	0.115 (6)	0.019 (2)	0.093 (5)	0.007 (3)	0.085 (5)	0.000 (3)
C10	0.091 (4)	0.0167 (18)	0.051 (3)	−0.005 (2)	0.052 (3)	−0.0055 (17)
C11	0.127 (6)	0.028 (2)	0.027 (2)	−0.016 (3)	0.040 (3)	−0.0104 (17)
C12	0.101 (5)	0.023 (2)	0.028 (2)	−0.017 (2)	0.013 (3)	−0.0027 (16)
C13	0.146 (7)	0.031 (3)	0.034 (3)	−0.028 (4)	−0.007 (4)	0.002 (2)
C14	0.126 (7)	0.053 (4)	0.062 (4)	−0.045 (4)	−0.045 (5)	0.017 (3)
C15	0.063 (4)	0.057 (3)	0.079 (4)	−0.022 (3)	−0.015 (3)	0.023 (3)
C16	0.049 (3)	0.045 (3)	0.047 (3)	−0.016 (2)	0.006 (2)	0.005 (2)
C17	0.059 (3)	0.0224 (18)	0.028 (2)	−0.0147 (19)	0.011 (2)	0.0014 (15)
C18	0.039 (2)	0.0197 (17)	0.0168 (16)	0.0072 (16)	0.0052 (15)	−0.0013 (13)
C19	0.035 (2)	0.0214 (17)	0.0191 (16)	0.0052 (15)	0.0000 (15)	0.0017 (13)
C20	0.029 (2)	0.0293 (19)	0.0221 (17)	0.0038 (16)	−0.0012 (16)	0.0035 (15)
C21	0.054 (3)	0.0222 (18)	0.0261 (19)	0.0072 (18)	−0.0128 (19)	−0.0028 (15)
C22	0.075 (4)	0.0229 (19)	0.0226 (19)	−0.002 (2)	−0.012 (2)	0.0012 (15)
C23	0.066 (3)	0.035 (2)	0.033 (2)	−0.005 (2)	−0.001 (2)	0.0079 (18)
C24	0.080 (4)	0.052 (3)	0.042 (3)	−0.017 (3)	0.009 (3)	0.010 (2)
C25	0.114 (6)	0.059 (4)	0.050 (3)	−0.029 (4)	0.001 (4)	0.022 (3)
C26	0.115 (6)	0.034 (3)	0.043 (3)	−0.016 (3)	−0.012 (3)	0.019 (2)
C27	0.087 (4)	0.025 (2)	0.034 (2)	0.004 (2)	−0.018 (3)	0.0059 (18)
C28	0.102 (5)	0.029 (2)	0.040 (3)	0.015 (3)	−0.017 (3)	0.003 (2)
C29	0.070 (4)	0.041 (3)	0.044 (3)	0.024 (3)	−0.026 (3)	−0.017 (2)
C30	0.090 (5)	0.055 (3)	0.068 (4)	0.038 (4)	−0.033 (4)	−0.012 (3)
C31	0.066 (4)	0.071 (4)	0.069 (4)	0.040 (4)	−0.023 (3)	−0.019 (3)
C32	0.057 (4)	0.089 (5)	0.058 (4)	0.017 (3)	−0.010 (3)	−0.028 (3)
C33	0.051 (3)	0.048 (3)	0.050 (3)	0.020 (2)	−0.011 (2)	−0.013 (2)
C34	0.056 (3)	0.034 (2)	0.036 (2)	0.014 (2)	−0.014 (2)	−0.0124 (18)
C35	0.044 (3)	0.032 (2)	0.080 (4)	0.022 (2)	0.007 (3)	0.005 (2)
C36	0.061 (4)	0.056 (4)	0.109 (6)	0.033 (3)	0.000 (4)	0.020 (4)
C37	0.076 (4)	0.051 (3)	0.068 (4)	0.023 (3)	−0.007 (3)	0.024 (3)
C38	0.056 (3)	0.042 (2)	0.0161 (17)	−0.004 (2)	0.0130 (18)	0.0007 (16)
C39	0.056 (3)	0.039 (2)	0.0193 (18)	−0.003 (2)	0.0113 (19)	−0.0079 (16)
C40	0.037 (2)	0.0282 (19)	0.0200 (18)	0.0001 (17)	0.0070 (16)	−0.0047 (14)
C41	0.044 (3)	0.036 (2)	0.034 (2)	−0.006 (2)	−0.002 (2)	−0.0020 (17)
C42	0.051 (3)	0.036 (2)	0.056 (3)	−0.014 (2)	0.005 (2)	−0.004 (2)
C43	0.057 (3)	0.027 (2)	0.042 (2)	−0.009 (2)	0.021 (2)	−0.0025 (17)
C44	0.140 (6)	0.023 (2)	0.049 (3)	−0.020 (3)	0.060 (4)	−0.012 (2)
C45	0.062 (3)	0.0257 (19)	0.040 (2)	−0.014 (2)	0.032 (2)	−0.0065 (17)
C46	0.054 (3)	0.031 (2)	0.042 (3)	0.002 (2)	−0.023 (2)	−0.0043 (18)
C47	0.064 (3)	0.0226 (19)	0.028 (2)	0.003 (2)	−0.011 (2)	−0.0035 (15)
C48	0.051 (3)	0.035 (2)	0.056 (3)	0.006 (2)	−0.007 (2)	−0.004 (2)
C49	0.100 (6)	0.077 (5)	0.125 (7)	0.032 (5)	−0.056 (6)	−0.029 (5)
C50	0.062 (4)	0.090 (6)	0.135 (8)	−0.007 (4)	0.035 (5)	−0.044 (5)
B1	0.057 (3)	0.026 (2)	0.0174 (19)	0.004 (2)	0.008 (2)	0.0094 (16)

### Geometric parameters (Å, º)

**Table:**

Eu1—O1	2.351 (3)	C20—C46	1.515 (5)
Eu1—O2	2.313 (3)	C21—C22	1.418 (7)
Eu1—O3	2.344 (3)	C21—C34	1.399 (7)
Eu1—O4	2.340 (3)	C22—C23	1.411 (8)
Eu1—O5	2.417 (3)	C22—C27	1.443 (6)
Eu1—N1	2.582 (3)	C23—H23	0.9500
Eu1—N3	2.555 (3)	C23—C24	1.364 (7)
Eu1—N5	2.573 (3)	C24—H24	0.9500
O1—C1	1.251 (5)	C24—C25	1.410 (9)
O2—C3	1.258 (5)	C25—H25	0.9500
O3—C18	1.266 (5)	C25—C26	1.339 (10)
O4—C20	1.261 (5)	C26—H26	0.9500
O5—C48	1.223 (6)	C26—C27	1.434 (9)
N1—N2	1.357 (5)	C27—C28	1.359 (9)
N1—C40	1.333 (5)	C28—H28	0.9500
N2—C38	1.339 (5)	C28—C29	1.393 (9)
N2—B1	1.549 (6)	C29—C30	1.411 (9)
N3—N4	1.359 (4)	C29—C34	1.455 (7)
N3—C41	1.330 (6)	C30—H30	0.9500
N4—C43	1.340 (6)	C30—C31	1.348 (11)
N4—B1	1.538 (6)	C31—H31	0.9500
N5—N6	1.346 (5)	C31—C32	1.422 (10)
N5—C35	1.326 (6)	C32—H32	0.9500
N6—C37	1.343 (6)	C32—C33	1.374 (8)
N6—B1	1.542 (7)	C33—H33	0.9500
N7—C48	1.312 (7)	C33—C34	1.407 (8)
N7—C49	1.452 (9)	C35—H35	0.9500
N7—C50	1.431 (10)	C35—C36	1.374 (9)
C1—C2	1.421 (5)	C36—H36	0.9500
C1—C45	1.507 (5)	C36—C37	1.380 (10)
C2—C3	1.413 (5)	C37—H37	0.9500
C2—C4	1.495 (5)	C38—H38	0.9500
C3—C44	1.522 (6)	C38—C39	1.374 (6)
C4—C5	1.409 (6)	C39—H39	0.9500
C4—C17	1.402 (6)	C39—C40	1.387 (5)
C5—C6	1.408 (7)	C40—H40	0.9500
C5—C10	1.445 (6)	C41—H41	0.9500
C6—H6	0.9500	C41—C42	1.383 (7)
C6—C7	1.370 (7)	C42—H42	0.9500
C7—H7	0.9500	C42—C43	1.374 (7)
C7—C8	1.427 (10)	C43—H43	0.9500
C8—H8	0.9500	C44—H44A	0.9800
C8—C9	1.335 (10)	C44—H44B	0.9800
C9—H9	0.9500	C44—H44C	0.9800
C9—C10	1.423 (9)	C45—H45A	0.9800
C10—C11	1.377 (9)	C45—H45B	0.9800
C11—H11	0.9500	C45—H45C	0.9800
C11—C12	1.372 (9)	C46—H46A	0.9800
C12—C13	1.430 (9)	C46—H46B	0.9800
C12—C17	1.436 (6)	C46—H46C	0.9800
C13—H13	0.9500	C47—H47A	0.9800
C13—C14	1.347 (11)	C47—H47B	0.9800
C14—H14	0.9500	C47—H47C	0.9800
C14—C15	1.409 (11)	C48—H48	0.9500
C15—H15	0.9500	C49—H49A	0.9800
C15—C16	1.366 (8)	C49—H49B	0.9800
C16—H16	0.9500	C49—H49C	0.9800
C16—C17	1.429 (7)	C50—H50A	0.9800
C18—C19	1.409 (5)	C50—H50B	0.9800
C18—C47	1.513 (5)	C50—H50C	0.9800
C19—C20	1.402 (5)	B1—H1	1.0000
C19—C21	1.512 (5)		
			
O1—Eu1—O5	75.01 (11)	C22—C21—C19	118.8 (4)
O1—Eu1—N1	145.99 (10)	C34—C21—C19	121.1 (4)
O1—Eu1—N3	142.63 (10)	C34—C21—C22	120.0 (4)
O1—Eu1—N5	118.84 (11)	C21—C22—C27	119.9 (5)
O2—Eu1—O1	70.86 (9)	C23—C22—C21	122.8 (4)
O2—Eu1—O3	145.67 (9)	C23—C22—C27	117.3 (5)
O2—Eu1—O4	86.42 (12)	C22—C23—H23	118.8
O2—Eu1—O5	109.01 (12)	C24—C23—C22	122.3 (5)
O2—Eu1—N1	140.38 (10)	C24—C23—H23	118.8
O2—Eu1—N3	79.35 (11)	C23—C24—H24	120.0
O2—Eu1—N5	72.80 (11)	C23—C24—C25	120.1 (7)
O3—Eu1—O1	79.81 (9)	C25—C24—H24	120.0
O3—Eu1—O5	79.12 (10)	C24—C25—H25	119.9
O3—Eu1—N1	73.13 (9)	C26—C25—C24	120.3 (6)
O3—Eu1—N3	117.69 (11)	C26—C25—H25	119.9
O3—Eu1—N5	139.36 (10)	C25—C26—H26	119.1
O4—Eu1—O1	80.32 (11)	C25—C26—C27	121.8 (5)
O4—Eu1—O3	71.10 (9)	C27—C26—H26	119.1
O4—Eu1—O5	144.11 (10)	C26—C27—C22	118.2 (6)
O4—Eu1—N1	109.05 (11)	C28—C27—C22	119.1 (5)
O4—Eu1—N3	75.70 (10)	C28—C27—C26	122.7 (5)
O4—Eu1—N5	143.07 (11)	C27—C28—H28	118.6
O5—Eu1—N1	79.92 (11)	C27—C28—C29	122.8 (4)
O5—Eu1—N3	137.76 (10)	C29—C28—H28	118.6
O5—Eu1—N5	72.66 (11)	C28—C29—C30	123.2 (6)
N3—Eu1—N1	70.18 (10)	C28—C29—C34	119.0 (5)
N3—Eu1—N5	70.74 (11)	C30—C29—C34	117.8 (6)
N5—Eu1—N1	73.60 (11)	C29—C30—H30	119.4
C1—O1—Eu1	137.5 (2)	C31—C30—C29	121.2 (6)
C3—O2—Eu1	139.3 (3)	C31—C30—H30	119.4
C18—O3—Eu1	135.7 (2)	C30—C31—H31	118.9
C20—O4—Eu1	135.2 (3)	C30—C31—C32	122.3 (6)
C48—O5—Eu1	134.9 (3)	C32—C31—H31	118.9
N2—N1—Eu1	125.2 (2)	C31—C32—H32	121.1
C40—N1—Eu1	128.1 (3)	C33—C32—C31	117.9 (7)
C40—N1—N2	105.9 (3)	C33—C32—H32	121.1
N1—N2—B1	121.4 (3)	C32—C33—H33	118.9
C38—N2—N1	110.1 (3)	C32—C33—C34	122.3 (6)
C38—N2—B1	128.4 (3)	C34—C33—H33	118.9
N4—N3—Eu1	125.2 (2)	C21—C34—C29	119.1 (5)
C41—N3—Eu1	128.2 (3)	C21—C34—C33	122.3 (4)
C41—N3—N4	106.1 (3)	C33—C34—C29	118.6 (5)
N3—N4—B1	122.4 (3)	N5—C35—H35	124.6
C43—N4—N3	109.8 (3)	N5—C35—C36	110.9 (6)
C43—N4—B1	127.7 (4)	C36—C35—H35	124.6
N6—N5—Eu1	125.4 (2)	C35—C36—H36	127.6
C35—N5—Eu1	126.3 (3)	C35—C36—C37	104.8 (5)
C35—N5—N6	106.6 (4)	C37—C36—H36	127.6
N5—N6—B1	121.6 (3)	N6—C37—C36	107.8 (5)
C37—N6—N5	109.9 (4)	N6—C37—H37	126.1
C37—N6—B1	128.4 (4)	C36—C37—H37	126.1
C48—N7—C49	119.4 (7)	N2—C38—H38	125.8
C48—N7—C50	122.5 (6)	N2—C38—C39	108.5 (4)
C50—N7—C49	118.0 (7)	C39—C38—H38	125.8
O1—C1—C2	125.6 (4)	C38—C39—H39	127.7
O1—C1—C45	116.1 (3)	C38—C39—C40	104.5 (4)
C2—C1—C45	118.2 (3)	C40—C39—H39	127.7
C1—C2—C4	120.3 (3)	N1—C40—C39	110.9 (4)
C3—C2—C1	120.5 (3)	N1—C40—H40	124.5
C3—C2—C4	119.2 (3)	C39—C40—H40	124.5
O2—C3—C2	124.9 (4)	N3—C41—H41	124.5
O2—C3—C44	116.1 (4)	N3—C41—C42	111.0 (4)
C2—C3—C44	119.1 (4)	C42—C41—H41	124.5
C5—C4—C2	120.4 (4)	C41—C42—H42	127.7
C17—C4—C2	120.1 (4)	C43—C42—C41	104.6 (4)
C17—C4—C5	119.5 (4)	C43—C42—H42	127.7
C4—C5—C10	118.9 (5)	N4—C43—C42	108.5 (4)
C6—C5—C4	123.2 (4)	N4—C43—H43	125.7
C6—C5—C10	117.9 (5)	C42—C43—H43	125.7
C5—C6—H6	118.8	C3—C44—H44A	109.5
C7—C6—C5	122.3 (5)	C3—C44—H44B	109.5
C7—C6—H6	118.8	C3—C44—H44C	109.5
C6—C7—H7	120.5	H44A—C44—H44B	109.5
C6—C7—C8	119.0 (7)	H44A—C44—H44C	109.5
C8—C7—H7	120.5	H44B—C44—H44C	109.5
C7—C8—H8	119.7	C1—C45—H45A	109.5
C9—C8—C7	120.7 (6)	C1—C45—H45B	109.5
C9—C8—H8	119.7	C1—C45—H45C	109.5
C8—C9—H9	118.9	H45A—C45—H45B	109.5
C8—C9—C10	122.1 (6)	H45A—C45—H45C	109.5
C10—C9—H9	118.9	H45B—C45—H45C	109.5
C9—C10—C5	118.0 (6)	C20—C46—H46A	109.5
C11—C10—C5	120.0 (5)	C20—C46—H46B	109.5
C11—C10—C9	122.0 (5)	C20—C46—H46C	109.5
C10—C11—H11	119.0	H46A—C46—H46B	109.5
C12—C11—C10	122.0 (4)	H46A—C46—H46C	109.5
C12—C11—H11	119.0	H46B—C46—H46C	109.5
C11—C12—C13	122.9 (5)	C18—C47—H47A	109.5
C11—C12—C17	119.0 (5)	C18—C47—H47B	109.5
C13—C12—C17	118.1 (6)	C18—C47—H47C	109.5
C12—C13—H13	118.9	H47A—C47—H47B	109.5
C14—C13—C12	122.2 (6)	H47A—C47—H47C	109.5
C14—C13—H13	118.9	H47B—C47—H47C	109.5
C13—C14—H14	120.1	O5—C48—N7	124.7 (6)
C13—C14—C15	119.7 (6)	O5—C48—H48	117.6
C15—C14—H14	120.1	N7—C48—H48	117.6
C14—C15—H15	119.5	N7—C49—H49A	109.5
C16—C15—C14	120.9 (7)	N7—C49—H49B	109.5
C16—C15—H15	119.5	N7—C49—H49C	109.5
C15—C16—H16	119.5	H49A—C49—H49B	109.5
C15—C16—C17	121.1 (6)	H49A—C49—H49C	109.5
C17—C16—H16	119.5	H49B—C49—H49C	109.5
C4—C17—C12	120.6 (5)	N7—C50—H50A	109.5
C4—C17—C16	121.5 (4)	N7—C50—H50B	109.5
C16—C17—C12	117.9 (5)	N7—C50—H50C	109.5
O3—C18—C19	124.9 (3)	H50A—C50—H50B	109.5
O3—C18—C47	115.2 (3)	H50A—C50—H50C	109.5
C19—C18—C47	120.0 (3)	H50B—C50—H50C	109.5
C18—C19—C21	118.2 (3)	N2—B1—H1	109.9
C20—C19—C18	122.0 (3)	N4—B1—N2	109.6 (3)
C20—C19—C21	119.8 (3)	N4—B1—N6	109.7 (3)
O4—C20—C19	125.1 (3)	N4—B1—H1	109.9
O4—C20—C46	115.7 (4)	N6—B1—N2	107.9 (4)
C19—C20—C46	119.3 (3)	N6—B1—H1	109.9
