# Supplementary material for: Bis[3-(anthracen-9-yl)pentane-2,4-dionato-κ2 O,O′](N,N-di­methyl­formamide-κO)[tris­(pyrazol-1-yl-κN 2)hydroborato]europium(III)
Source: Acta Crystallogr E Crystallogr Commun. 2022 Jan 28;78(Pt 2):103–7. doi: 10.1107/S2056989022000676 (PMC8819440; doi:10.1107/S2056989022000676)
Supplement: Supplementary file 3 [file e-78-00103-sup3.pdf]

Supplementary Material, Part-2

Bis(3-[9'-anthracenyl]pentane-2,4-dionato)(*N,N*-dimethylformamide-O)(tris[pyrazol-1-yl-κN(2)]hydroborato)europium(III)

Elena A. Mikhalyova,* Matthias Zeller, Jerry P. Jasinski, Manpreet Kaur, Anthony W. Addison

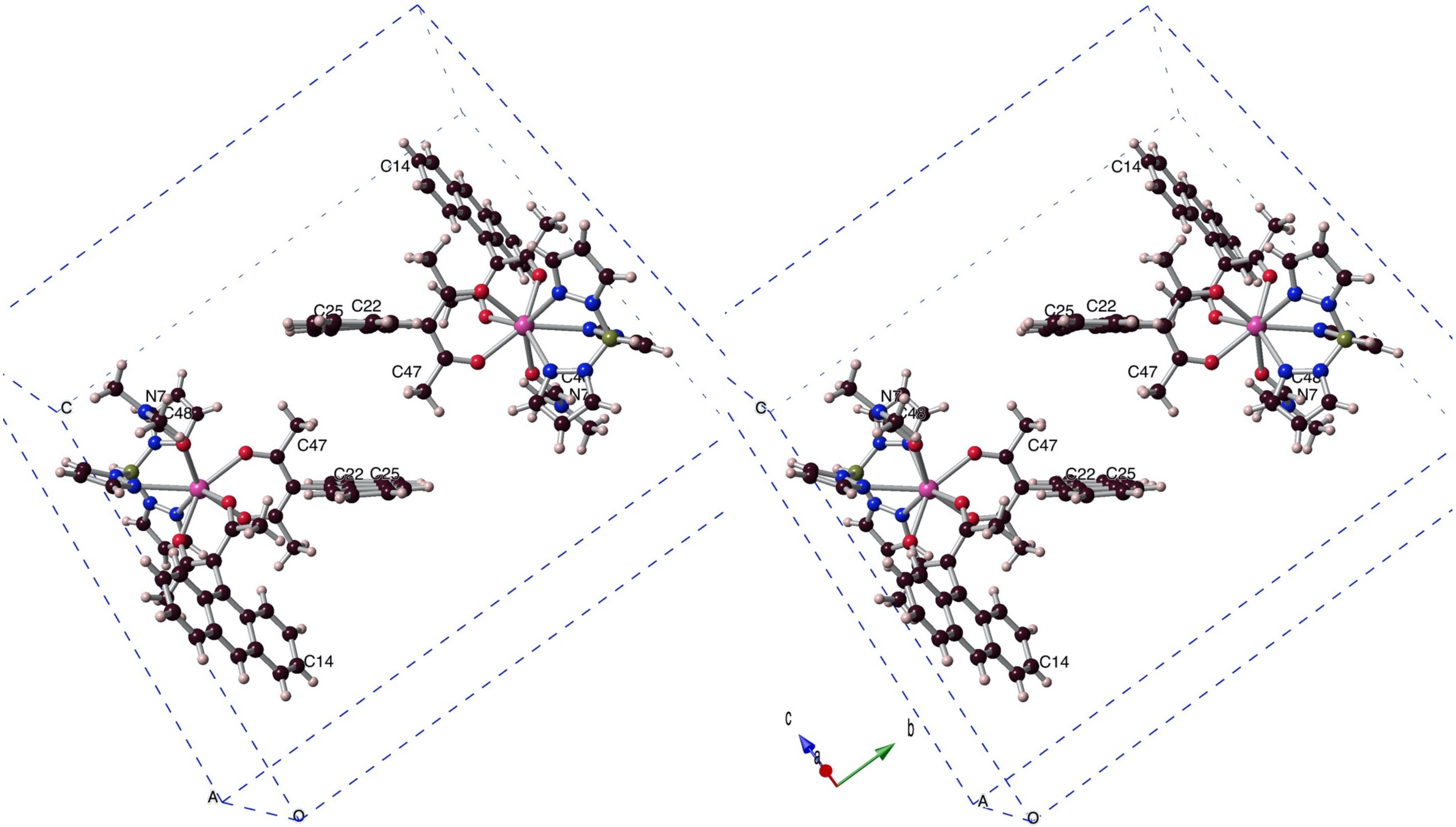

Figure S1: An illustration of the intervention of the C47 methyl group, in the centre of the figure, between the anthracene rings above and below it containing carbon atoms C21-C34 (ball & stick model, inverse stereoview).

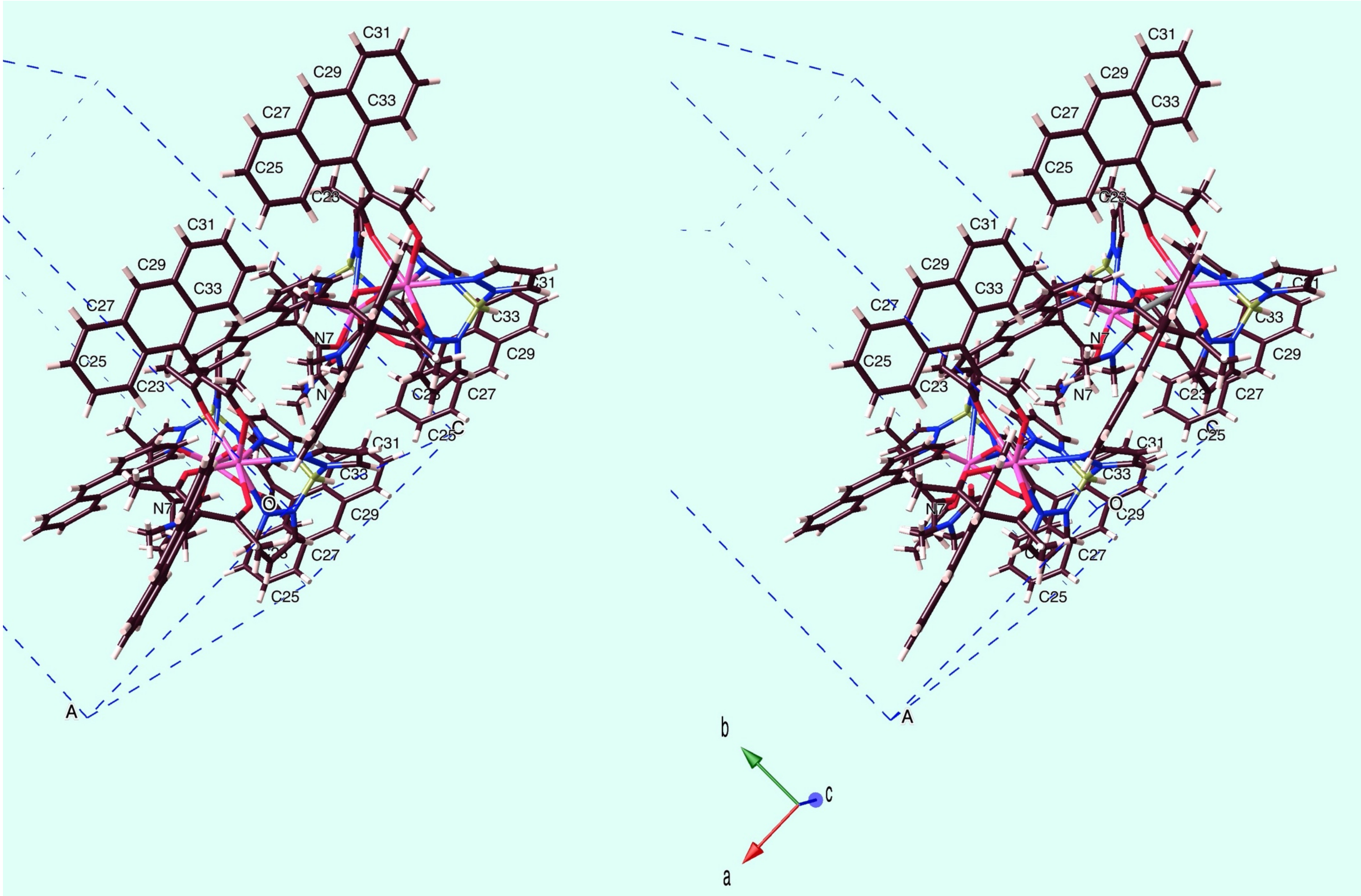

Figure S2: An alternative pairing of anthracene rings C21-C34 (upper left), indicating how the offset nature of this particular interaction also disallows π-stacking (stick model, inverse stereoview).

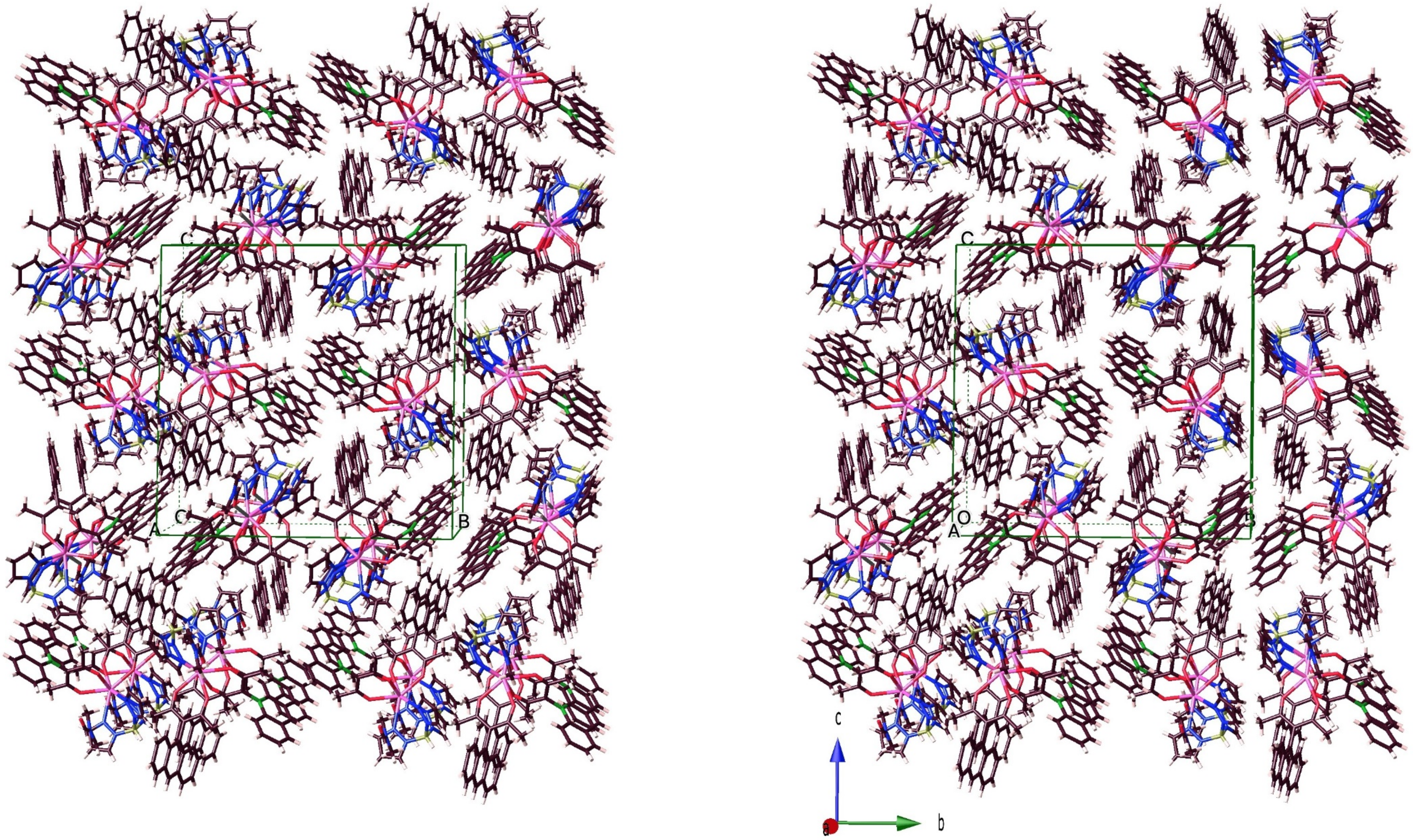

Figure S3: Lattice packing diagram, showing how a view obliquely along the *a*-direction depicts ordering which is less apparent in other views of the unit cell (stick structure, inverse stereoview).

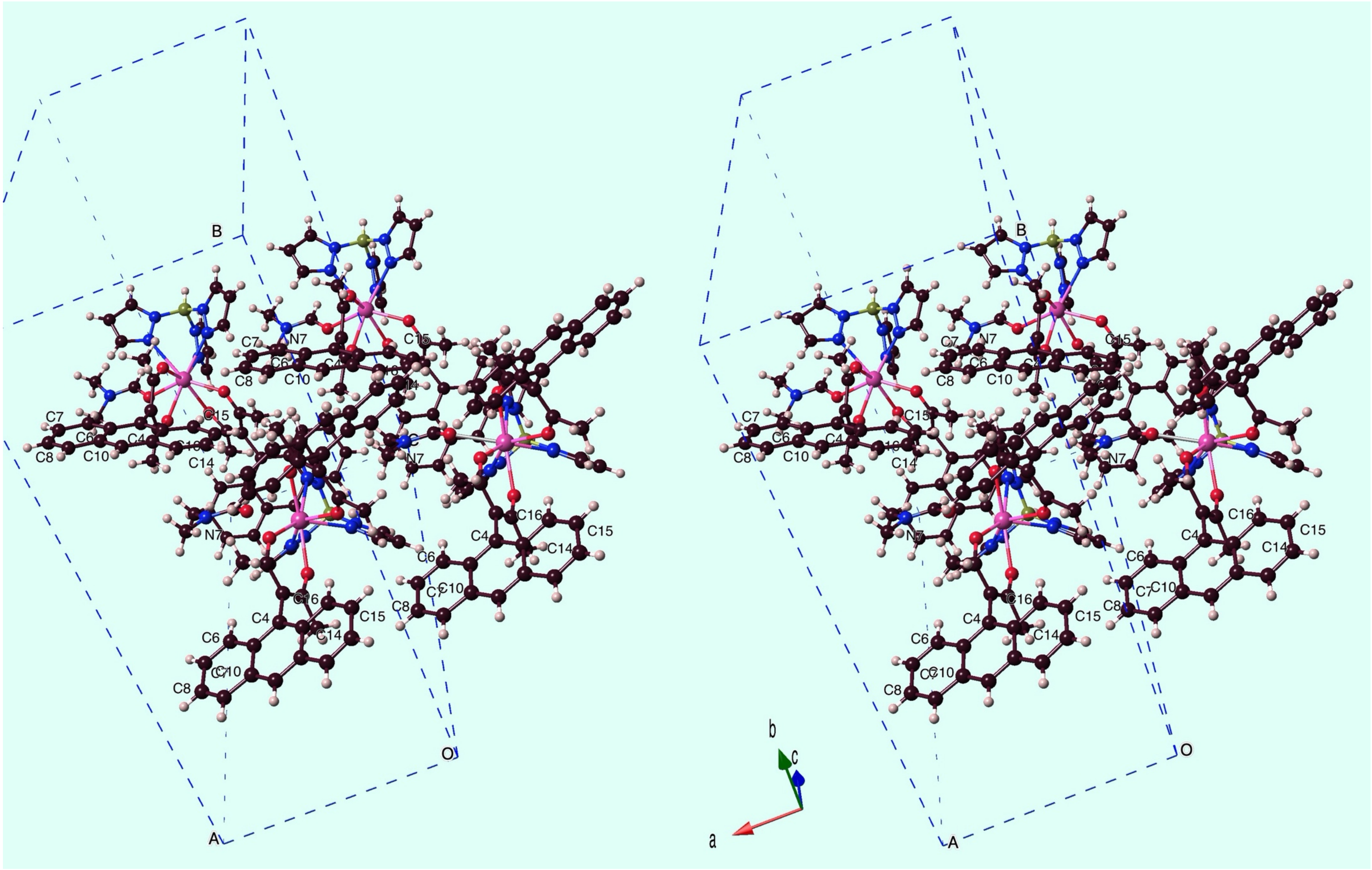

Figure S4: A lattice fragment illustrating how the offset nature of the parallel anthracene rings C4-C17 (lower right) thwarts any π-π stacking between them (ball & stick model, inverse stereoview).
